# Experimental Studies on Interfacial Shear Characteristics between Polypropylene Woven Fabrics

**DOI:** 10.3390/ma12223649

**Published:** 2019-11-06

**Authors:** Fu Yi, Hui Li, Jia Zhang, Xutong Jiang, Maocheng Guan

**Affiliations:** 1 College of Architecture and Transportation, Liaoning Technical University, Fuxin 123000, China; yifu9716@163.com; 2 China Coal Research Institute, Beijing 100013, China; 3 School of Civil Engineering, Liaoning Technical University, Fuxin 123000, China; 4 Institute of Urban Rail Transit, Liaoning Railway Vocational and Technical College, Jinzhou 121000, China; 5 Institute of Railway Engineering, Liaoning Railway Vocational and Technical College, Jinzhou 121000, China; zhangjia19850504@126.com

**Keywords:** interfacial shear characteristic, pull-out test, interfacial types, dry-wet state, sliding friction angle, occlusal friction angle

## Abstract

Geotextile tubes are used in dam construction because fine tailings are difficult to use. The shear characteristics of geotextile tubes during dam operation are closely related to those of the materials used to construct the tubes. Pull-out tests can accurately reflect the interfacial shear characteristics between geosynthetics in practice, so pull-out tests were carried out for different interfacial types of polypropylene woven fabrics under dry and wet states. The effects of the type of interface and dry-wet states on the interfacial shear characteristics were investigated, and the impact mechanisms were also discussed. The results indicated that P-type interfaces (the warp yarn on the interface is parallel to the pulling direction) tended to harden. However, PTP-type (the warp yarn on the interface is perpendicular to each other) and T-type (the weft yarn on the interface is parallel to the pulling direction) interfaces softened first and then tended to plateau after reaching peak shear stress, and softening became more obvious at higher normal stresses. The displacement corresponding to peak shear stress (referred to as “peak displacement” in this paper) of interfaces was positively correlated with the normal stress, and the wet state reduced the interfacial peak displacement. For different types of interfaces, the peak displacement of the T-type interface was the largest, followed by PTP-type and P-type. Interfacial shear characteristics conformed to Mohr–Coulomb strength theory and, compared with quasi-cohesion values ranging from 1.334 to 3.606 kPa, the quasi-friction angle significantly contributed to the interfacial shear strength. The quasi-friction angle of the interface was composed of a sliding friction angle and an occlusal friction angle. The shear strength of the interface was more sensitive to the interface types than whether they were in the dry or wet state. For different types of interfaces and dry-wet states, the change in the interfacial shear strength is respectively affected by the occlusal friction angle and the sliding friction angle on the interface.

## 1. Introduction

With the rapid development of mining technology in recent years, mineral processing technology and resource recovery ratios have continuously improved. The amount of tailings discharged into tailings reservoirs has significantly increased because of this, and tailings particles have become increasingly finer [1,2,3,4]. Fine-grained tailings cannot be used in dam construction by an upstream method due to their poor mechanical characteristics [5]. Simultaneously, with increased awareness of environmental protection, the exploitation of sand and gravel resources will become severely restricted, and the high transportation costs of sand and gravel will increase the construction cost of dams. Therefore, the use of fine-grained tailings locally to construct and guarantee the stability of dams should be investigated.

Geosynthetics have been successfully used to enhance structural stability in various engineering applications [6]. Geotextile tubes with high length-width ratios are made of geosynthetics with certain tensile strengths and are filled with tailings slurries. The consolidated tailings and geotextiles form composite soils with different strengths. In recent years, geotextile tubes have been widely used in dam construction [7], reinforced embankment [8], land reclamation [9], shoreline management [10] and sludge dewatering [11]. The use of geotextile tubes to construct dams can overcome the difficulties in dam construction due to fine tailings. Compared with traditional earth–rock dams, geotextile tube dams have several advantages, including reduced energy consumption, environmental protection, simple construction process, low cost, weather resistance, and a well-defined construction period. New dams rely on shear characteristics between geotextile tubes to maintain their stability. Many authors have conducted significant amounts of research on the shear characteristics of geotextile tube-geotextile tube interfaces and tailings-geotextile interfaces to rapidly develop this technology [12,13,14,15,16,17,18]. However, the shear characteristics between geotextile tube interfaces are closely related to those of the tailings, the interface between geotextiles, and tailings–geotextile interfaces [19]. When geotextile tubes are stacked layer-by-layer, they are directly in contact with each other. The destruction of geotextile tube dams mainly arises due to sliding along the interface between geotextiles. Therefore, investigating the interfacial shear characteristics between geotextiles can provide important parameters for dam designs, which are also important for dam stability analyses.

Carbone [20] investigated the dry friction characteristics for a non-woven geotextile–geomembrane interface using inclined plane tests and shaking table tests. Aldeeky [21] discussed the effects of sand placement on the shear characteristics of interfaces of sand-woven geotextiles and sand non-woven geotextiles on an inclined plane at different angles. Bacas [22] performed direct shear tests to study the shear interaction mechanisms of three interfaces, including geotextile-geomembranes, drainage geocomposite-geomembranes, and soil-geomembranes used in landfills. For the first two interfaces, the interfacial shear strength was proportional to the asperity height, whereas for the soil–geomembrane interface, higher soil shear strengths led to higher interface shear strengths. Beliaev [23] noted that the static friction coefficient was different by up to 20% for interfaces between aramid fabrics with different types of weaving. Aiban [24] used pull-out tests to conduct experimental research on the frictional characteristics of sand–geotextile–sand and sabkha–geotextile–sand interfaces. It was found that the strain on the interface gradually decreased from the pull end to the free end, and the interfacial friction characteristics were affected by the surface texture and geotextile permeability. Yang [19] investigated the friction characteristics of the interface between polypropylene geotextiles through direct shear tests and noted that friction characteristics conformed to the Mohr–Coulomb strength theory. Huang [25] conducted comparative research on the interface shear characteristics between sand-woven fabrics and sand-burst film yarn using pull-out tests. It was reported that compared with the burst film yarn, woven fabrics provided better interlocked structures, which led to a more pronounced interface hardening. Yin [26] explored the shear characteristics of fine tailing–geotextile interface and fine tailing–geogrid interface by pull-out tests in the laboratory, and found that the interfacial friction coefficient did not exceed 0.22. Pan [27] studied the shear characteristics of filling sand, geotextiles–geotextiles interface, sand–geotextile interface, and geotextile tube–geotextile tube interface by direct tests and pull-out tests. The results showed that the quasi-friction angle for the geotextile tube–geotextile tube interface was most similar to the quasi-friction angle average of filling sand and the interface between geotextiles.

**Figure 1:**
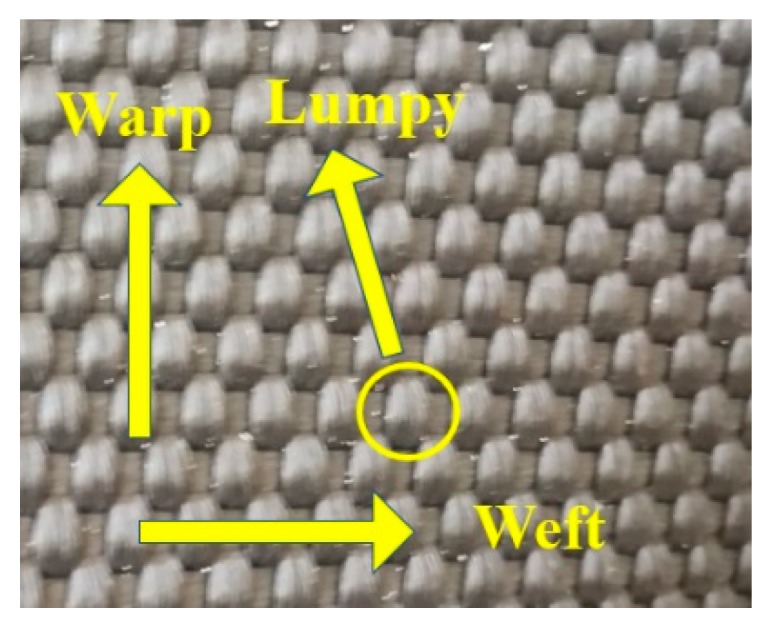
Surface of woven polypropylene fabric.

Although the above results have provided valuable guidance for the design of geotextile tubes, there is still much that needs to be further investigated. Firstly, the woven fabrics used for geotextile tube construction are woven by warp and weft, and the material surface is slightly lumpy (Figure 1). Different warp and weft combinations between woven fabrics will affect the interfacial shear characteristics [28]. Secondly, due to geotextile tube drainage, woven fabrics will inevitably become wet with water, and the interfacial shear characteristics under dry and wet conditions must be studied separately. Furthermore, compared with direct shear tests, pull-out tests can fully demonstrate the effect of occlusion on the interface and better restore the practical development of pull-out forces [29].

Therefore, to investigate the interfacial shear characteristics between woven fabrics, this paper is devoted to experimental studies on the interfacial shear characteristics for different types of interfaces under both dry and wet conditions using pull-out tests. The effects of interfacial types and dry–wet states on the relationship between the pull-out force and pull-out displacement, peak displacement, and interfacial shear strength are reported, and the influence mechanisms are discussed.

## 2. Materials and Methods 

### 2.1. Test Materials

Geotextile tubes are inevitably exposed to complex environmental variations including sunlight, rain washes, temperature changes, etc. Geotextiles used to construct geotextile tubes must conform to requirements for drainage, sand retention, strength, and durability. Generally, polypropylene woven fabrics and burst film yarn warp knitted geotextiles are the main raw materials used in geotextile tubes. Compared with the latter, the former conforms better with the requirements mentioned above and has better friction characteristics [30]. Therefore, in this paper, polypropylene woven fabrics were used in pull-out tests to investigate the interfacial shear characteristics. The parameters of polypropylene woven fabrics are summarized in Table 1.

### 2.2. Test Equipment

**Figure 2:**
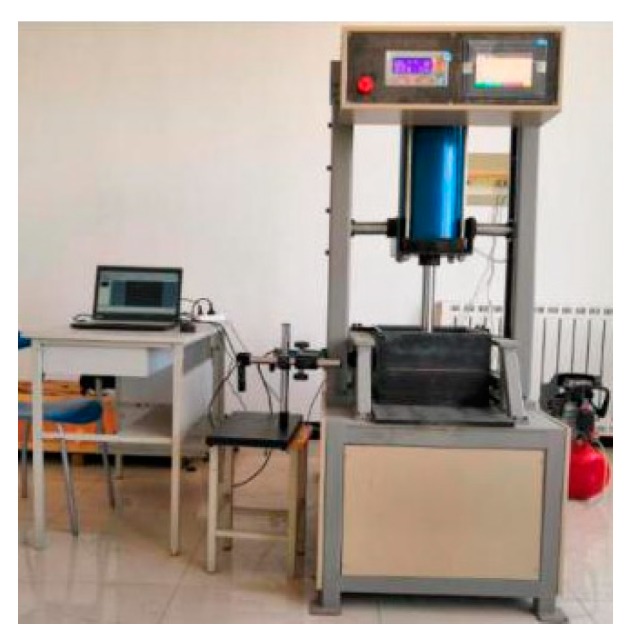
The pull-out test equipment.

Pull-out tests utilized a YT1200 geosynthetics direct shear and pull-out test system produced by Nanjing Huade Instrument Co., Ltd (Nanjing, China). The equipment was composed of four parts: a pull-out box, a vertical loading system, a horizontal loading system, and a data acquisition system. The total size of the pull-out box inner wall was 300 × 300 × 220 mm, and a narrow seam of 300 × 10 mm was reserved both in the front center and back of the pull-out box. The vertical-loading system formed from a cylinder with a pressure transducer provided a normal stress varying from 0 to 200 kPa through a bearing plate with a size of 300 × 300 × 10 mm. The horizontal-loading system containing a tension and compression motor and a tension sensor provided a constant loading rate which ranged from 0 to 5 mm/min within 100 mm and also simultaneously measured the pull-out force. The pull-out test equipment is shown in Figure 2.

### 2.3. Test Program

**Figure 3:**
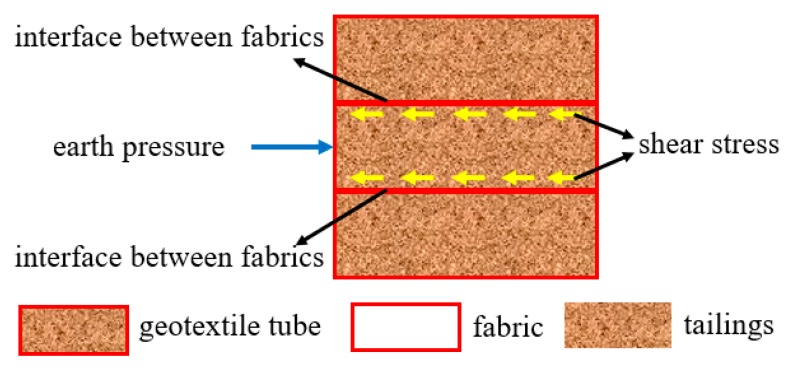
Force diagram of the interface between geotextile tubes.

During the operation of geotextile tube dams, the earth pressure from the tailings deposited in tailings reservoirs act on geotextile tubes. Due to the shear stress acting on the interface of geotextile tubes, the earth pressure was resisted to maintain the stability of the dam, as shown in Figure 3. The geotextile tubes interface was mainly contact between woven fabrics, and so the interfacial shear characteristics of the fabrics directly affected those of the geotextile tubes.

In order to restore the mechanical characteristics of woven fabrics in geotextile tube dams, pull-out tests between woven fabrics were carried out using the equipment shown in Figure 2. In the tests, three pieces of woven fabrics were stacked horizontally, and a vertical load system was used to apply normal stresses to the interfaces. When a horizontal pulling force was applied to the middle woven fabric, the fabric slid along the upper and lower interfaces in a manner similar to the sliding of geotextile tubes in practical applications. 

Since the pulling fixture was fixed at the same level as the middle of the pull-out seam, the pulled woven fabric had to be placed in the middle of the pull-out seam to horizontally pull it. To satisfy this condition, we chose to fill the tailings into the pull-out box to provide a highly suitable platform for the tests. Although the above condition was met, when the lower woven fabric was directly laid on the tailings, it wrinkled due to the pulling force, which led to a change in the interface area during the tests. Even worse, it may have been pulled out along with the middle woven fabric. Therefore, a 300 × 300 mm piece of woven fabric was pasted to both a prefab 300 × 300 × 20 mm steel plate and the bearing plate to guarantee a constant interface area during the tests.

To prepare the test platform, a pull-out box whose inner wall was evenly smeared with lubricant was filled and compacted with tailings layer-by-layer to prevent the effect of the compression deformation of the tailings on the pulling force direction. The height of the tailings filled into the pull-out box was kept at about 50 mm each time, and a normal stress of 150 kPa was applied to the tailings through the bearing plate and compacted for 10 minutes. The filling and compaction process was repeated until the tailings surface was 15 mm below the bottom edge of the pull-out seam when the normal stress for compacting was removed.

**Figure 4:**
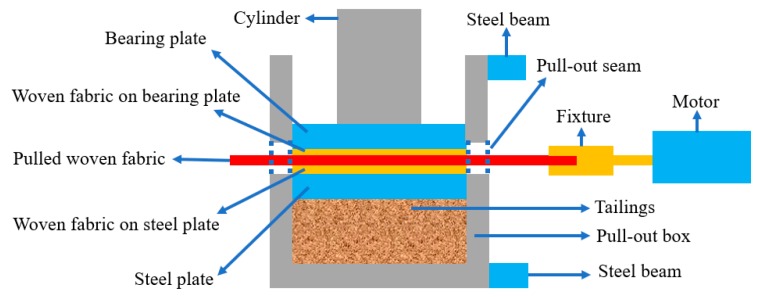
Schematic of the pull-out test.

Then, the prefab steel plate was placed on the tailings surface with the woven fabric facing upward. A 500 × 300 mm woven fabric was laid on the steel plate using the front and back pull-out seams. One end of the woven fabric was clamped with the fixture in the horizontal loading system, and the other end remained free. Then, normal stresses of 25 kPa, 50 kPa, 75 kPa, and 100 kPa were applied by a vertical-loading system, and after that, the woven fabric in the middle was horizontally pulled at a rate of 2 mm/min through a horizontal-loading system. Finally, the pull-out forces and displacements were recorded every 3 s using a data acquisition system which was built into the YT1200 system, and the curves reflecting the relationship between the pull-out forces and displacements were displayed synchronously. The pull-out test schematic diagram is shown in Figure 4.

Woven fabrics are anisotropic materials. When filling geotextile tubes, the fabrics were inevitably wetted due to discharged water. Furthermore, during the operation of geotextile tube dams, geotextile tubes under the seepage line were immersed in water for a long time. Woven fabrics provided different friction coefficients in dry and wet conditions. Therefore, in this paper, different warps and wefts were combined under different dry and wet states to form 6 kinds of interfaces, as summarized in Table 2 where upper stands for the woven fabric pasted on the bearing plate, middle stands for the pulled woven fabric, and lower stands for the woven fabric pasted on the prefab steel plate. When the interface was wetted, the letter W appeared in the interfacial code, otherwise, the letter D appeared.

Since the interfaces were located within range of the pull-out seam, it was not possible to perform underwater pull-out tests. To simulate the immersion of the interface in water, three layers of woven fabrics were immersed for 24 h and then taken out prior to tests and quickly pasted to the prefab steel plate and the bearing plate.

Pull-out tests were carried out in strict accordance with Test Methods of Geosynthetics for Highway Engineering (JTG E50–2006) used in China. In the test, the contact area of the upper, middle, and lower samples was constant at 0.18 m^2^. To reduce the error of test results, three sets of parallel tests were performed in each group of tests, and the average value was used in calculations.

## 3. Results

### 3.1. The Effect of Interfacial Type and Dry–Wet State on the Characteristics of Shear Stress and Pull-Out Displacement Curves 

**Figure 5:**
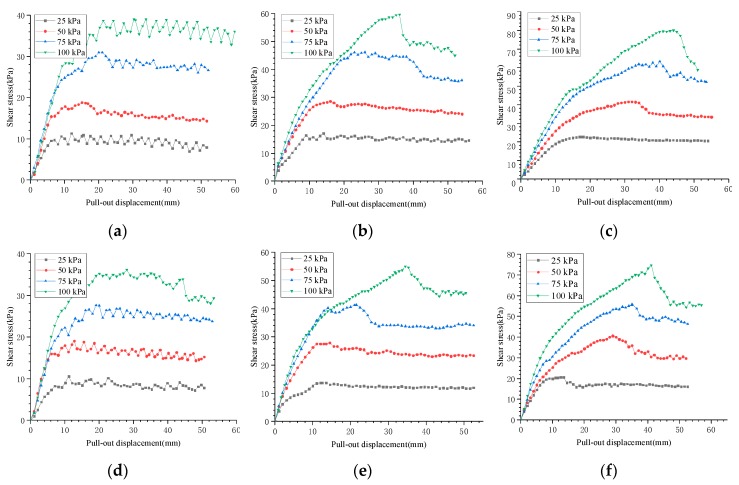
Shear stress and pull-out displacement curves. (**a**) The curves on P-D interface. (**b**) The curves on PTP-D interface. (**c**) The curves on T-D interface. (**d**) The curves on P-W interface. (**e**) The curves on PTP-W interface. (**f**) The curves on T-W interface.

The relationship between the shear stress and pull-out displacement for six interfaces is shown in Figure 5. It can be seen that the shapes of these curves under different interfacial types and dry-wet states were generally the same, but contained several differences. At the initial stage of the pull-out test (about 0 to 10 mm pull-out displacement), the shear stress rapidly increased approximately linearly with the pull-out displacement. Then, the growth rate decreased significantly, and the curve showed a distinct non-linear shape. The dry-wet state had almost no effect on the shape of shear stress and pull-displacement curves, while the normal stress and interfacial type had significant influences.

**Figure 6:**
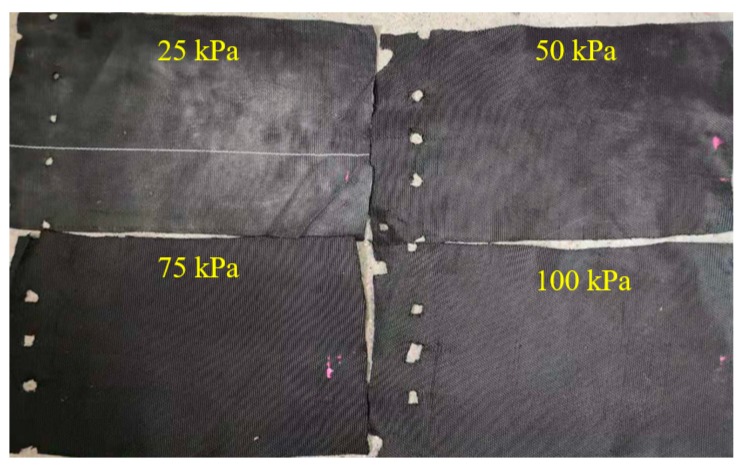
P-type interface after the pull-out test.

For the P-type (the warp yarn on the interface is parallel to the pulling direction) interface, the peak shear stress was not obvious, whether in the dry or wet state, and the shear stress and the pull-out displacement curves showed obvious hardening. When the pull-out displacement was small (within about 5 mm), the shear stress rapidly and linearly increased. When the shear stress was near its peak, the curves began to show zigzag fluctuations. The shear stress decreased slightly after reaching the peak shear stress, and the magnitude of the shear stress reduction was positively correlated with the normal stress at the interface. The analysis of the residual strength can provide significant experimental evidence for the design of geotextile tube dams [31]. The residual strength at a pull-out displacement of 50 mm (hereafter referred to as “large displacement strength”) reached about 90% of the shear strength. This indicated that the strength loss rate of the P-type interface was lower when larger deformation occurred. The P-type interface after the pull-out test is shown in Figure 6.

Figure 6 shows that, for the P-type interface, due to pulling along the warp and because the warp strength was larger than the weft strength, no obvious damage occurred at the contact between the fixture and pulled woven fabric. Thus, no obvious trace of interface deformation was observed in the interface after tests.

For PTP-type (the warp yarn on the interface is perpendicular to each other) and T-type (the weft yarn on the interface is parallel to the pulling direction) interfaces, the shear stress and the pull-out displacement curves varied with the normal stress acting on the interface. When the normal stress was 25 kPa, peak shear stress was not obvious, and obvious hardening of the curve was observed. It was found that the above two interfaces maintained high strength under large deformations, and the large displacement strength also accounted for about 90% of the shear strength. As the normal stress increased, the peak shear stress became increasingly obvious, and the curve indicated that softening occurred before plateauing. After reaching the peak shear stress, the shear stress decreased more rapidly with the pull-out displacement, and the decrease was greater at higher normal stresses. The shear stress decreased slowly and gradually stabilized as the displacement continued to increase. The large displacement strength reached 70%–85% of the shear strength. 

During pull-out tests, pulling forces on interfaces were gradually transmitted from the clamping end to the free end. For different interfacial types, dry–wet states, and normal stresses, the peak shear stresses occurred at different displacements depending on the difficulty of transmitting the pulling force. For example, the greater the normal stress was, the more difficult the transmitting process was, and the more time was needed for the pulling force to reach its peak. Therefore, for the same interface, the peak displacement significantly increased with the normal stress under a constant pull-out speed. In Section 4, the above phenomenon will be further discussed from the perspective of interfacial type and dry–wet state.

### 3.2. The Effect of Interfacial Type and Dry–Wet State on Pull-Out Displacement

**Figure 7:**
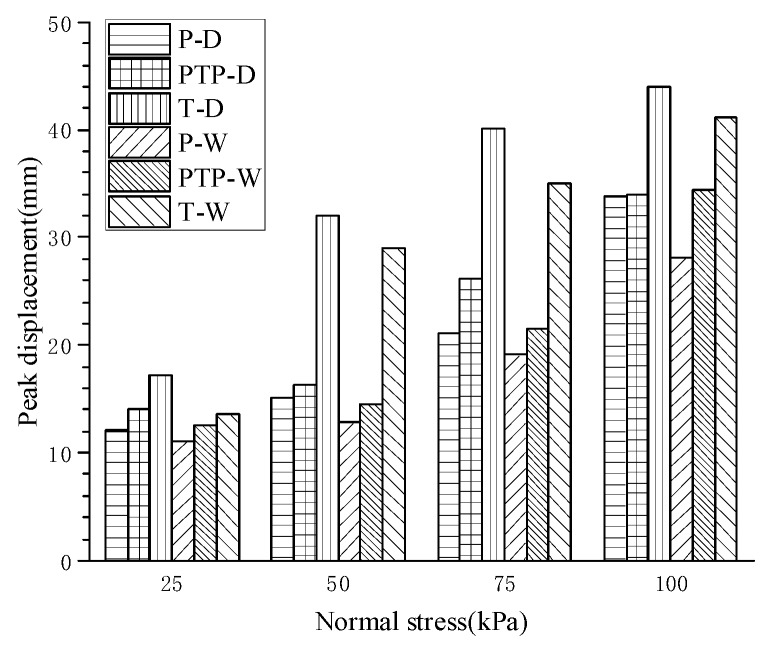
Relationship between peak displacement and normal stress for different interfaces.

The peak displacements for 6 interfaces in Table 2 under different normal stresses were obtained from Figure 5. The relationships between the normal stress and peak displacement of different interfaces are plotted in Figure 7. It is obvious that the interfacial peak displacement was affected by the normal stress, interfacial type, and dry–wet state. For the same interface, the peak displacement significantly increased with the normal stress. When the interfacial types were the same, the peak displacement of the dry interface was larger than that of the wet interface. Under the same dry–wet state, the T-type interface showed the largest peak displacement, followed by PTP-type and P-type. Furthermore, the peak displacement of the T-type interface was the most sensitive to normal stress, whether in the dry or wet sate. 

Before reaching the peak pulling force, the displacement of pulled woven fabric decreased from the clamping end to the free end, and the displacement mainly manifested as tensile deformation of the woven fabric. When the pulling force reached its peak, the displacement was transmitted to the free end, and the entire pulled woven fabric began to slide. Therefore, there were two displacement components: tensile deformation of pulled woven fabric (before peak displacement) and overall sliding displacement (after peak displacement). As can be seen from Figure 5, the shear characteristics in the same interface were different in the stage of tensile deformation and the stage of overall sliding. In the stage of tensile deformation, shear stress increased with an increase of tensile deformation. In overall sliding stage, the interface experienced varying degrees of softening which was positively correlated with normal stress acting on interfaces.

**Figure 8:**
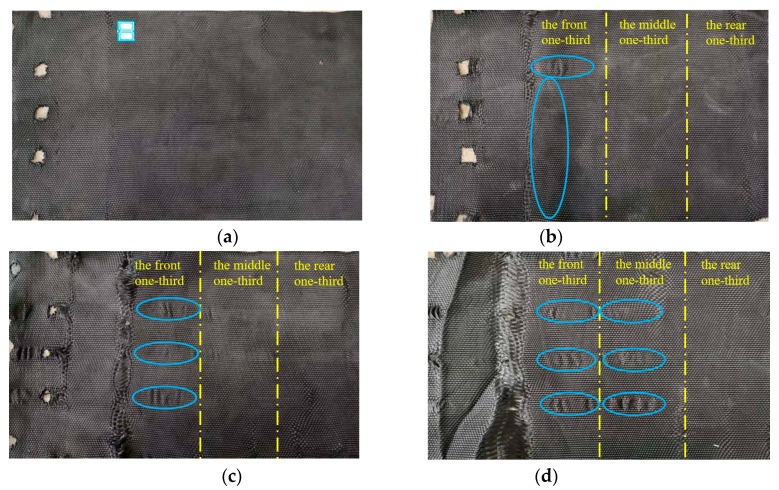
T-type interface after the pull-out test. (**a**) 25 kPa normal stress. (**b**) 50 kPa normal stress. (**c**) 75 kPa normal stress. (**d**) 100 kPa normal stress.

The T-type interface after the pull-out test is shown in Figure 8. Comparing Figure 6 and Figure 8, as the normal stress increased at the T-type interface, the damage at the contact between the fixture and pulled woven fabric became increasingly obvious, and traces of tensile deformation at the interface became clearer. The blue marks indicate traces after the tensile deformation of the woven fabrics. The tensile deformation of the interface could be divided into three different areas. The closer it was to the clamping end, the larger the tensile deformation was, and the clearer the deformation trace was. The above phenomenon was consistent with the results in reference [24].

### 3.3. The Effect of Interfacial Type and Dry–Wet State on the Interfacial Shear Strength

**Figure 9:**
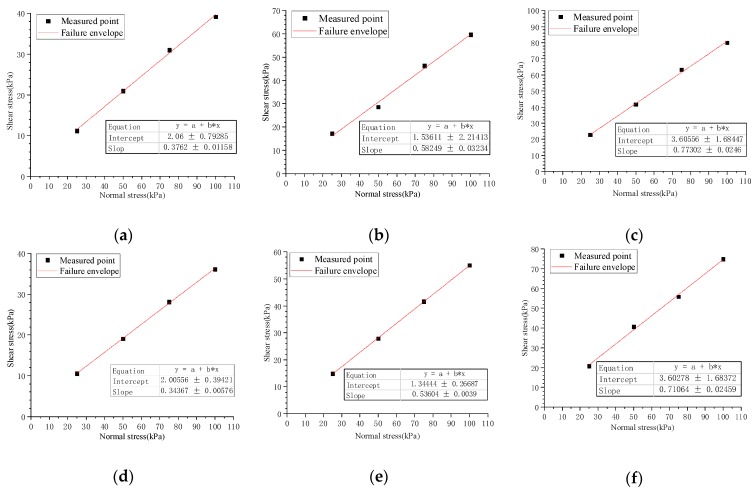
Failure envelopes of shear strengths of different interfaces. (**a**) Failure envelope of shear strength on P-D interface. (**b**) Failure envelope of shear strength on PTP-D interface. (**c**) Failure envelope of shear strength on T-D interface. (**d**) Failure envelope of shear strength on P-W interface. (**e**) Failure envelope of shear strength on PTP-W interface. (**f**) Failure envelope of shear strength on T-W interface.

The peak shear stresses of each interface in Table 2 were obtained under each normal stress from Figure 5 and are summarized in Table 3. The linear fitting of the normal stresses and peak shear stresses were used to obtain the failure envelopes of shear strength for different interfaces (Figure 9), whose correlation coefficients were all greater than 0.99. This indicated that the interfacial shear strengths conformed to Mohr–Coulomb strength theory. Therefore, quasi-friction angle and quasi-cohesion were selected as the indexes to reflect the interfacial shear characteristics which are summarized in Table 3. When both the normal stress and dry-wet state were identical, the T-type had the largest interfacial shear strength, PTP-type was second and P-type was the smallest. Compared with the wet interface, the dry interface offered a larger interfacial shear strength at the same interface type and normal stress. Additionally, under the same normal stress, the sensitivity of interfacial shear strength to the interfacial type was higher than the dry–wet state. Additionally, the higher the normal stress, the greater the sensitivity.

**Figure 10:**
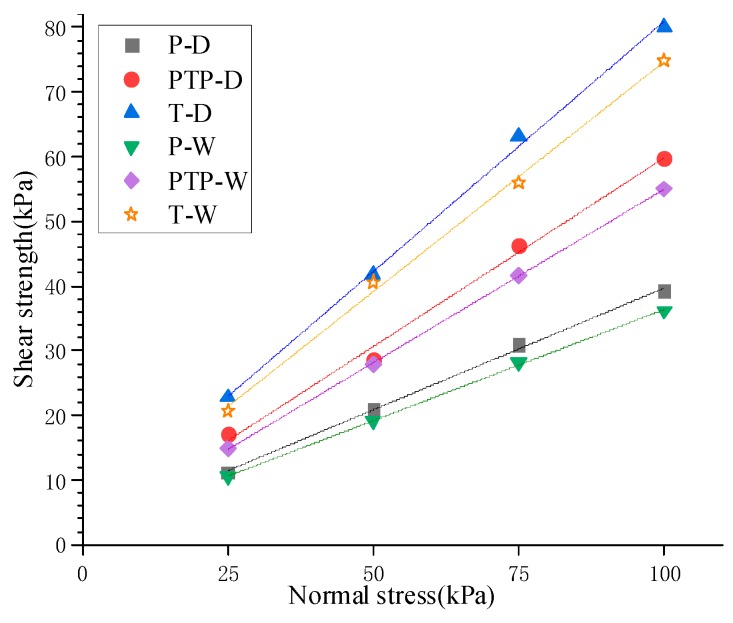
Comparison plot of failure envelopes of the shear strengths of different interfaces.

By plotting these failure envelopes on the same figure in Figure 10, the fitting curves were approximately regarded as many lines with different slopes scattered from the same point. The ordinate of the point where lines originated was equivalent to the interfacial quasi-cohesion, and the slopes of these lines corresponded to interfacial quasi-friction coefficient, which was tangent to the interfacial quasi-friction angle. 

**Figure 11:**
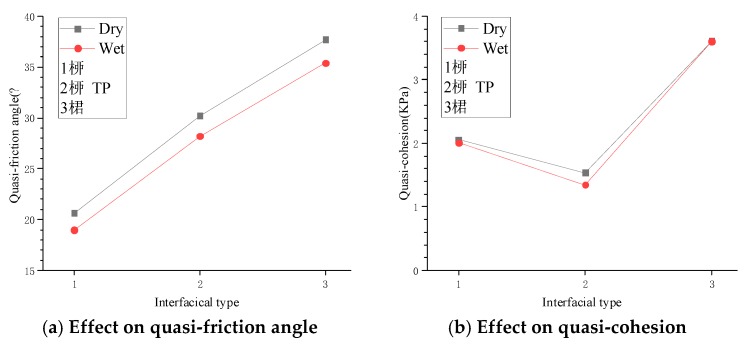
Effects of the interfacial type and dry–wet state on the interfacial shear strength indexes.

To investigate the effect of interfacial type and dry-wet state on the indexes of interfacial shear strength, the relationships between the interfacial shear strength indexes and interfacial type under different dry–wet states were plotted in Figure 11. 

For P-type, PTP-type, and T-type interfaces in the dry state, the quasi-friction angles were 20.616°, 30.22°, and 37.704°, respectively, while they were 18.968°, 28.191°, and 35.393° in the wet state. The wet interface decreased the quasi-friction angles by 8.0%, 6.7%, and 6.1% for the three interfaces above, respectively. The effect of the wet state on the quasi-friction angle in the P-type interface was slightly larger than the others. Compared with P-type and PTP-type interfaces, in the dry state, the quasi-friction angle of the T-type interface increased by 82.9% and 24.8%, while in the wet state it increased by 86.6% and 25.5%. It was revealed that although the quasi-friction angle varied with both the interfacial type and dry–wet state, changing the dry–wet state only slightly influenced the quasi-friction-like angle. In contrast, changing the type of interface significantly changed the quasi-friction angle.

Figure 11b shows that the dry–wet state hardly affected the quasi-cohesion for the same type of interface. Although the quasi-cohesion was sensitive to the interfacial types, compared with the dry-wet state, it was still very small and varied from 1.334 to 3.606 kPa.

Therefore, it can be concluded that changing the quasi-friction angle significantly contributed to the different interfacial shear strengths due to different types of interfaces and dry–wet states, despite that both of the indexes affected the interfacial shear strength. That was to say, changing the interfacial type and dry–wet state was essentially expressed as a change in the quasi-friction angle.

## 4. Discussion

Based on the above analyses, it was found that the interfacial shear characteristics between woven fabrics were closely related to the interfacial type and dry–wet state. Each of these factors primarily affected the interfacial shear characteristics through the quasi-friction angle. This section is focused on the quasi-friction angle to explore the influence mechanism of the interfacial type and dry–wet state on the interfacial shear characteristics between woven fabrics. Additionally, it seeks to reveal the underlying causes of experimental phenomena.

Friction angles included the sliding friction angle and the occlusal friction angle [32]. The former was determined by the type of materials, while the latter was controlled by interlocking action. Interlocking structures appeared at the interfaces when three layers of woven fabrics with slightly lumpy surfaces were tightly pressed, as shown in Figure 1 and Figure 4. The transmission of pulling forces had to overcome both the sliding friction between materials and the interlocking force provided by the interlocking structure.

### 4.1. Influence Mechanism of Dry–Wet State

Although the interfacial types were different, under the same normal stress the sliding friction forces acting on the interfaces were almost identical between the dry–wet states. Compared with the dry state, the resistance to the pulling force transmission decreased due to the lubrication of water molecules in the wet state, which decreased the peak displacement, shear strength, and quasi-friction angle. Therefore, the influence mechanism of the dry–wet state on interfacial shear characteristics was macroscopically represented by a decrease in the sliding friction angle in the wet sate. Since polypropylene is hydrophobic [33], the water molecules had less of a lubricating effect, and the sliding friction angle did not greatly decrease, which was consistent with Figure 11a.

### 4.2. Influence Mechanism of Interfacial Type

The influence of the type of interface on the interfacial shear characteristics was greater than that of the dry-wet state. Different types led to different stabilities between interfacial interlocking structures, which provided a large difference in interfacial interlocking forces. As mentioned in Section 3.1, P-type and T-type interfaces with the largest differences in interfacial shear characteristics were used as examples. 

**Figure 12:**
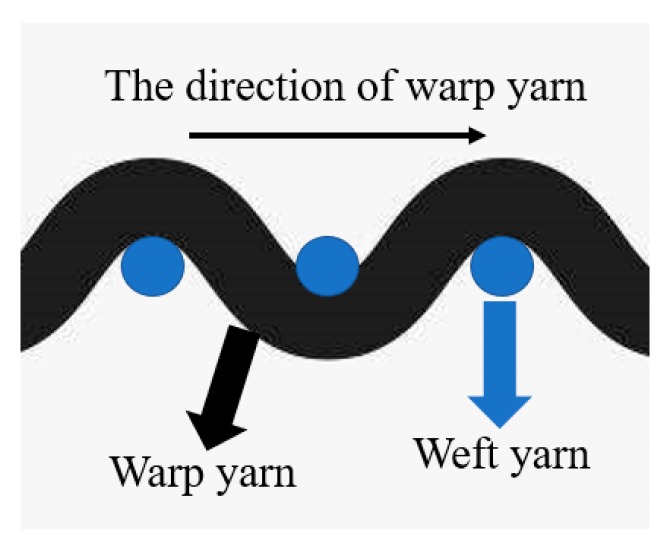
Cross-section along the warp yarn direction.

The displacement direction of the T-type interface was perpendicular to the warp yarn. When the interface was relatively displaced, the warp yarn of different woven fabrics contacted and blocked each other, as shown in Figure 12. At this time, for the interface to be pulled, the resistance between the warp yarn needed to be overcome, and the tighter the interface was pressed, the greater the resistance was. The resistance between the warp yarn was equivalent to the development of an interlocking structure which increased the occlusal friction. Under the same sliding friction force, the T-type interface showed the most stable interlocking structure and offered the highest shear strength compared with the other two interfaces (Figure 10).

However, the displacement direction of the P-type interface was parallel to warp yarn. Since the weft yarn of woven fabrics was not lumpy (Figure 1), the resistance between the weft yarn was too small to form a stable interlocking structure. Therefore, the shear strength of the P-type interface was mainly provided by the sliding friction force, giving it the smallest shear strength, as shown in Figure 10. The influence mechanism of the type of interface on the interfacial shear characteristics was macroscopically manifested by an increase in the occlusal friction angle when the interfacial type was changed from P-type to T-type. The values of the occlusal angle in the PTP-type and T-type interfaces were approximately equal to the increases of the quasi-friction angle compared with that of the P-type. Moreover, when the P-type interface was pulled by the entire piece, the movement along the raised warp yarn showed an undulating structure, as shown in Figure 12. Pulling uphill was difficult and the pulling force became larger. In contrast, the pulling force became smaller when pulled downhill. Therefore, after the peak pulling force was reached, the shear stress displayed zigzag fluctuations as the displacement increased, as shown in Figure 5a,d.

In summary, to improve the stability of geotextile tube dams, the warp yarn of woven fabrics should be oriented parallel to the dam length when sewing geotextile tubes. In this way, the T-type interface between geotextile tubes will be subjected to the earth pressure provided by tailings in the reservoir. Furthermore, the strength of the warp yarn should as high as possible, and its protrusions on the surface of the woven fabric should be strengthened to enhance the interlocking structure between woven fabrics.

## 5. Conclusions

To explore the interfacial shear characteristics between polypropylene woven fabrics, interfacial pull-out tests were performed under different interfacial types and dry–wet states, and the following conclusions were drawn, which can provide a useful reference for the design and construction of geotextile tube dams.

- Interfacial shear characteristics between polypropylene woven fabrics are in accordance with Mohr–Coulomb strength theory. The interfacial shear strength was determined by the quasi-friction angle and quasi-cohesion. The former played a dominant role, while the latter varied within a small range from 1.334 to 3.606 kPa. Each of these two indexes were more sensitive to the interfacial type than the dry–wet state, and the quasi-cohesion showed an intense resistance to changes in the dry–wet state. The quasi-friction angle was provided by the sliding fiction angle and occlusal friction angle. The former was mainly influenced by the dry–wet state through the lubrication of water molecules, while the latter was predominantly controlled by the interfacial types with different interlocking structures. In the wet state, a decrease in the interfacial shear strength was macroscopically represented by a reduction in the sliding friction angle. The T-type interface provided the largest interfacial shear strength among the types of formed interfaces, which was attributed to a macroscopic increase in the occlusal friction angle.
- Both the interfacial type and normal stress significantly influenced the shape of the curve plotted by the shear stress and pull-out displacement, but the dry–wet state had only a small influence. The curves of the six interfaces discussed in this paper showed different hardening tendencies. For the P-type interface, hardening was the most obvious, and the curve displayed zigzag fluctuations after reaching the peak shear stress, and the large displacement strength reached 90% of the interfacial shear strength. For the PTP-type and T-type interfaces, the hardening degree of the curve was related to the normal stress. At loads less than 25 kPa, the hardening characteristics were similar to those of the P-type interface mentioned above. When the loads varied from 50 to 100 kPa, the two interfaces exhibited similar trends where they first softened and then tended to plateau. Softening was more obvious at higher normal stresses. Large displacement strengths accounted for 70%–85% of the interfacial shear strength.
- The peak displacement was connected with the interfacial type, dry–wet state, and normal stress, and was positively correlated with normal stress. When other conditions were identical, the peak displacement on the dry interface was larger than that on the wet interface. When the interfacial types were different, the peak displacements were in the order: T-type > PTP-type > P-type. Regardless of whether dry or wet, the peak displacement of the T-type interface was the most sensitive to normal stress.
- During the construction of geotextile tube dams, it is suggested that the warp yarn of geotextile tubes should be parallel to the length of dams. Additionally, the raised structure of the warp yarn on the woven fabric surface should be strengthened, so as to improve the stability of the dams.

## Figures and Tables

**Table 1:** Parameters of polypropylene woven fabrics.

Physical Index	Unit	Test Method	Number of Samples	Value
Mass per unit area	g/m^2^	ASTM (American Society for Testing and Materials) D 5261	5	398
Thickness	Mm	ASTM D 5261	5	1.7
Warp strength	KN/m	ASTM D 4595	5	72
Weft strength	KN/m	ASTM D 4595	5	57
Longitudinal elongation	%	ASTM D 4595	5	15
Latitudinal elongation	%	ASTM D 4595	5	12
Equivalent aperture*O*_90_	Mm	ASTM D 4751	5	0.08

**Table 2:** The 6 kinds of interfaces used in the pull-out tests.

Interfacial Code	Dry-wet State	Parallel to Pulling Direction		
		Upper	Middle	Lower
P-D	Dry	Warp	Warp	Warp
PTP-D	Dry	Warp	Weft	Warp
T-D	Dry	Weft	Weft	Weft
P-W	Wet	Warp	Warp	Warp
PTP-W	Wet	Warp	Weft	Warp
T-W	Wet	Weft	Weft	Weft

**Table 3:** Indexes of interfacial shear characteristics of different interfaces.

Interfacial Code	Normal Stress (kPa)	Peak Shear Stress (kPa)	Quasi-friction Angle (°)	Quasi-cohesion (kPa)	Correlation Coefficient
P-D	25	11.16	20.616	2.058	0.99716
	50	20.98			
	75	30.97			
	100	39.18			
PTP-D	25	17.12	30.22	1.536	0.99081
	50	28.58			
	75	46.31			
	100	59.76			
T-D	25	22.77	37.704	3.606	0.99697
	50	41.68			
	75	63.21			
	100	80.02			
P-W	25	10.53	18.986	2.006	0.99916
	50	19.09			
	75	28.16			
	100	36.15			
PTP-W	25	14.89	28.191	1.344	0.99402
	50	27.89			
	75	41.62			
	100	54.99			
T-W	25	20.70	35.393	3.602	0.99642
	50	40.64			
	75	55.89			
	100	74.84			
